# Supplementary figures and images for: Screening a Spliced Leader-Based Symbiodinium microadriaticum cDNA Library Using the Yeast-Two Hybrid System Reveals a Hemerythrin-Like Protein as a Putative SmicRACK1 Ligand
Source: Microorganisms. 2021 Apr 9;9(4):791. doi: 10.3390/microorganisms9040791 (PMC8070245; doi:10.3390/microorganisms9040791)

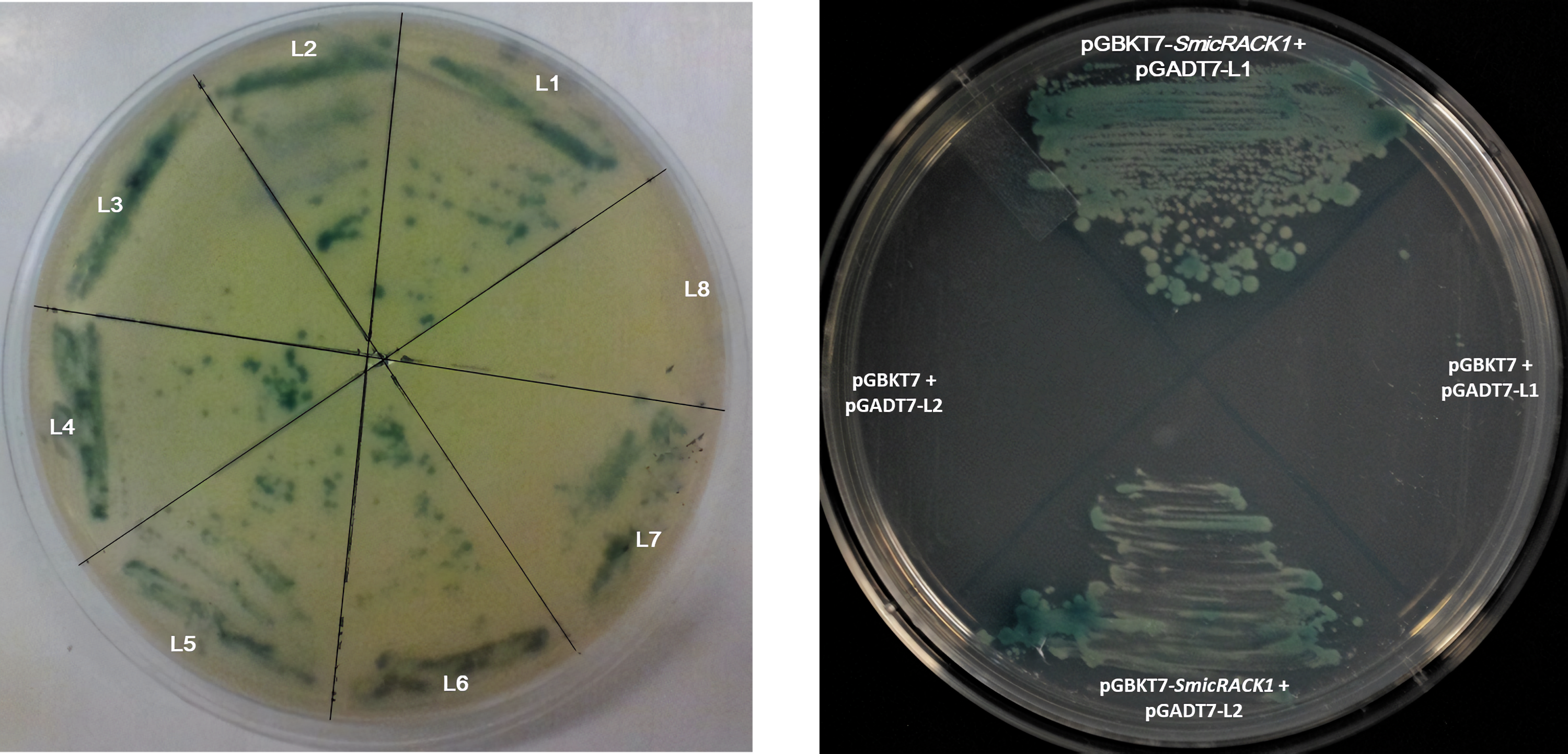

Supplement: Supplementary file 1 [file microorganisms-09-00791-s001.zip › Microorg-supp/Supplementary Figure 1.png]
